# Analgesic effect of Ropivacaine combined with Dexmedetomidine on brachial plexus block

**DOI:** 10.1186/s12871-018-0570-0

**Published:** 2018-08-16

**Authors:** Zhenqing Liu, Menglu Jiang, Tongsheng Xu, Hao Hua

**Affiliations:** Department of Anesthesiology, The Ninth People’s Hospital of Wuxi, No.999 Liangxi Road, Wuxi, 214062 China

**Keywords:** Dexmedetomidine, Ropivacaine, Nerve block, Analgesia, Visual analog scale

## Abstract

**Background:**

This randomized controlled study investigated the analgesic effect of ropivacaine in combination with dexmedetomidine versus ropivacaine alone on brachial plexus block to provide alternative anesthetic means for upper limb trauma surgery.

**Methods:**

Totally 114 patients who received upper limb surgeries under brachial plexus block anesthesia in our hospital from February 2013 to July 2015 were enrolled. The patients were randomized to ropivacaine alone (the control group) or ropivacaine combined with dexmedetomidine (the combination group). The blocking effect on sensory and motor neurons, visual analog scale (VAS) score, heart rate (HR), mean arterial pressure (MAP), peripheral capillary oxygen saturation (SPO_2_) and adverse reactions were compared between the two groups.

**Results:**

The time to onset of sensory and motor nerve blockade was significantly shorter in the combination group than in the control group (8.9 min vs. 12.4 min for sensation blockade; 7.5 min vs. 12.8 min for motor blockade, *P* < 0.05 for both comparisons), and the duration of the blockade was significantly longer in the combination group (590.2 min vs. 532.1 min, *P* < 0.05). There was no significant difference in VAS scores between the two groups immediately and 4 h after surgery; however, 8, 12 and 24 h after surgery, the VAS scores were all significantly lower in the combination group than the control group (2.4 vs. 3.0 for 8 h; 2.2 vs. 4.2 for 12 h, and 2.1 vs. 5.4 for 24 h, respectively, *P* < 0.05 for all comparisons). There was no statistical difference in HR, MAP and SPO_2_ between the two groups before anesthesia, but after anesthesia, the MAP and HR were significantly lower, and the SPO_2_ was significantly higher in the combination group than the control group (78 vs. 84 for MAP; 72 vs. 79 for HR; and 95.1 vs. 88.2 for SPO_2_, *P* < 0.05 for all comparisons). The rates of adverse reaction was significantly lower in the combination group than the control group (3.6 vs. 7.2, *P* < 0.05).

**Conclusion:**

The brachial plexus blocking effect of ropivacaine combined with dexmedetomidine was superior to that of ropivacaine alone, mainly intra-operatively and postoperatively.

**Trial registration:**

Analgesic Effect of Ropivacaine Combined with Dexmedetomidine on Brachial Plexus Block, ChiCTR1800017372, retrospectively registered on July 26, 2018.

## Background

With the dramatic increase in the number of automatic vehicles on the road, the incidence of various types of traffic traumas has been on the rise throughout China. Upper limb fracture is one of them. In order to alleviate severe pain in patients with upper limb fracture, analgesia by brachial plexus blockade is usually applied before surgery. However, due to a myriad of reasons, patients with upper limb fracture are usually very nervous and anxious before and during the operation, leading to heart rate (HR) decrease, blood pressure increase and even shock [1, 2].

Compared with general anesthesia, brachial plexus regional anesthesia is the preferred approach of anesthesia for upper limb surgery because it is easy to perform, and patients can undergo the procedure in the waking state during the operation [3]. However, administration of appropriate analgesics is critical [4]. Traditional analgesics such as fentanyl have strong analgesic effect; however, they were found to be prone to over-anesthesia; thus, they virtually increase perioperative risks and are not conducive to postoperative recovery [5]. At present, a technique of continuous brachial plexus blockade under the guidance of ultrasonography has been developed, where local anesthetics can be intermittently or continuously injected into the location to be blocked through a connection tube installed by visualization technique. This technique is very simple to use, and allows the adjustment of dose of the anesthetic according to operative time; it also meets postoperative analgesic requirement of patients [6].

In addition to the route of anesthesia, the choice of analgesics is also very important. An ideal analgesic is one that can achieve a good anesthetic effect without incurring serious adverse reactions. Ropivacaine is a new local anesthetic that inhibits neuronal excitement and conduction by inhibiting neuronal sodium channels [7]. It has a very strong analgesic effect as it can produce nerve blocking effect at low concentrations; its effect is long lasting, and its central nerve inhibitory activity is low, which makes it a commonly used anesthetic in nerve block anesthesia [8]. In addition, ropivacaine also has vasoconstrictive effect, thereby reducing absorption of drugs into the plasma and leading to a long lasting effect. The drug is also one of the ideal anesthetics to relieve a variety of postoperative pain. Dexmedetomidine is a highly selective α2 adrenergic receptor agonist. It shows a high affinity for its receptor that is 7 to 8 times stronger than clonidine, which belongs to the same category. It also shows a fast onset time and long lasting effect. Clinically, dexmedetomidine is used as an analgesic, sedative and anxiolytic medication [9]. Studies have shown that dexmedetomidine also has a neuroprotective effect, possibly by increasing intravascular calcium levels and reducing the concentration of catecholamines [10, 11]. Thus, dexmedetomidine has been as an anesthesia adjuvant.

In this study, we evaluated the efficacy and safety of ropivacaine in combination with dexmedetomidine versus ropivacaine alone in 114 patients requiring upper limb surgery with the primary outcome being changes in postoperative visual analog scale (VAS) scores versus baseline.

## Methods

### Patients

This randomized controlled study enrolled patients who were scheduled to undergo elective upper limb surgery, including tendon repair, fracture internal fixation, and flap transfer, at our hospital between February 2013 and July 2015. Patients were included 1) if they had an American Society of Anesthesiologists (ASA) physical status classification I or II; 2) if they were aged between 20 and 50 years; 3) if they had no known allergy to anesthetics; 4) if they had no history of heart, kidney and brain diseases. Patients were excluded 1) if they had chronic heart and lung diseases; 2) if they were obese (body mass index, BMI ≥ 30 kg/m^2^); 3) if they refused to provide consent to the study.

This study was approved by the local Institute Ethic Committee at the authors’ affiliated institution and all patients signed informed consent to voluntarily participate in the study.

### Randomization

Sample size calculation was based on the primary outcome of changes in postoperative VAS scores versus baseline. We calculated the sample size using the SAS Proc Power, and 48 patients for each group were required, assuming a two-sided type I error (*α*) of 0.05 and a power of 80%. Allowing for approximately 20% incomplete follow-up or dropout, a total of 120 patients were enrolled in this study.The patients were randomly assigned either to the control group or the combination treatment group in a 1:1 ratio using a computer-generated randomization number sequence performed by a clinician who was not involved in the study. Group assignment was sealed in sequentially numbered opaque envelopes. The patients, attending anaesthesiologists, surgeons, and data collectors were all blinded to patient group assignment.

### Brachial plexus block

Phenobarbital sodium (0.1 g) was administered intramuscularly 30 min before surgery. Immediately before the operation, oxygen was provided conventionally, an intravenous route was established and the vital signs including heart rate (HR), mean arterial pressure (MAP) and oxygen saturation (SpO2) were monitored conventionally. Brachial plexus block anesthesia was then started. Briefly, the patient was placed in a supine position, and the upper limbs were placed on the corresponding side of the body. The needle was inserted into the muscle layer at the circular cartilage level, and moved towards the posterolateral and lower direction of the grove. The needle was then turned slightly towards C6 cervical transverse process. When the stimulating electric current reached 0.5 mA, if the distal muscle of the deltoid muscle group contracted concurrently, the puncture was deemed successful. After the successful puncture, anesthetics were injected. The control group received 20 mL 0.375% ropivacaine within 30 s; the combination treatment group received 20 mL 0.375% ropivacaine in combination with 100 μg dexmedetomidine within 30 s.

### Study outcomes

During the whole procedure, vital signs of the patients were recorded. Pain was evaluated using the VAS, with 0 for no pain, and 10 for the worst possible pain. VAS scores at baseline (0 h), and 4, 8, 12 and 24 h after surgery were recorded. The primary outcome was changes in postoperative VAS scores versus baseline. The secondary outcomes included time to onset of blockade, time to recovery of sensation and movement and the total analgesic maintenance time, changes in HR, MAP and SPO_2_ and safety. Time to onset of blockade was defined as the time between injection of the anesthetics and loss of sensation to needle prick, and the time between injection of the anesthetics and loss of thumb movement. Time to onset of blockade of the radial nerve, median nerve, ulnar nerve and musculocutaneous nerve was recorded. In addition, time to recovery of sensation and movement and the total analgesic maintenance time were also recorded. Adverse reactions during the perioperative period were recorded.

### Statistical analysis

Data was expressed as mean ± SD and analyzed using the SPSS 13.0 software (SPSS Inc., Chicago, IL, USA). Comparison between the two groups was performed using Student’s *t* test. Count data were analyzed with χ^2^ test. *P* < 0.05 was considered statistically significant.

## Results

### Patient demographic and baseline data

**Fig. 1:**
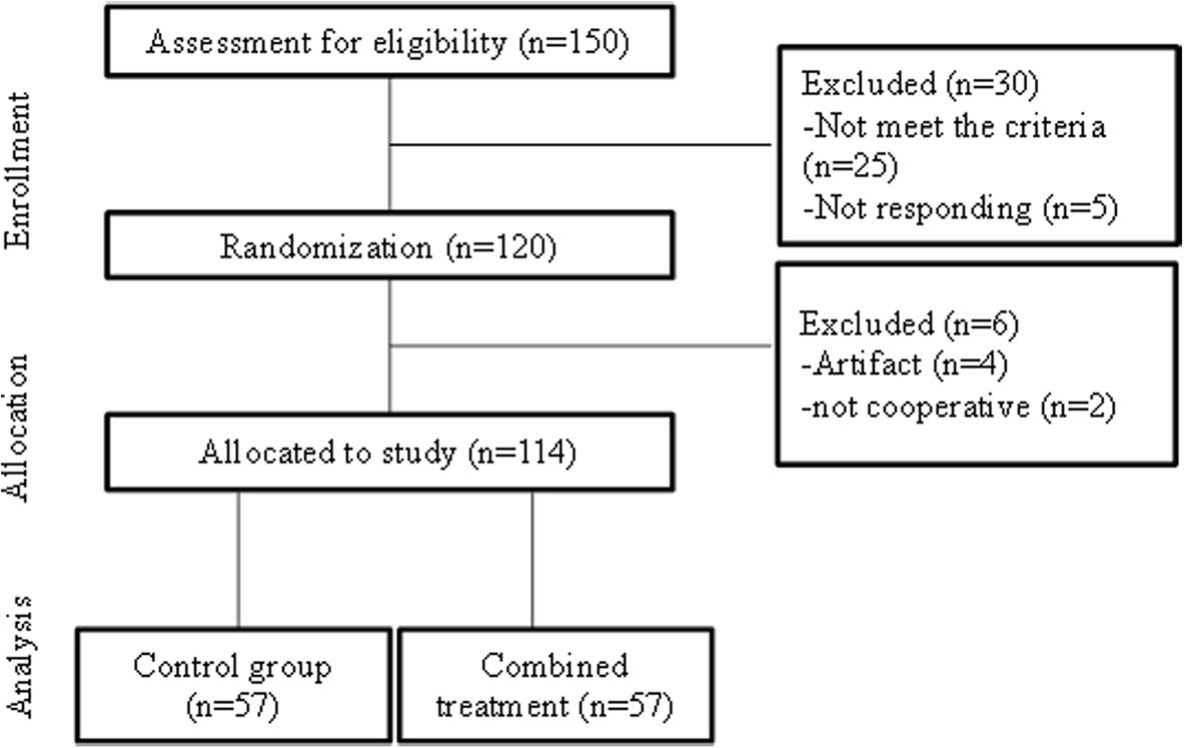
The study flowchart

The study flowchart is shown in Fig. 1. Totally 150 patients were screened for enrollment, 25 patients did not meet the eligibility requirements and were excluded, and 5 did not provide consent to the study and were not included. One hundred twenty patients were eligible; 6 patients were excluded because they were not cooperative during the study. Finally, 114 patients were included in the study. They included 37 (64.9%) male patients and 20 (35.1%) female patients in the combination treatment group and 36 (63.2%) male patients and 21 (36.8%) female patients in the control group. The age of patients in the combination treatment group (30.4 ± 10.1 years; range 20 to 55 years) was comparable with that of the control group (30.8 ± 11.2 years; range 21 to 55 years). The two groups were comparable in demographic variables (*P* > 0.05).

### Ropivacaine in combination with dexmedetomidine is more effective than ropivacaine in alleviating postoperative pain

The VAS scores of the two groups before and at different time points after surgery are summarized in Table 1. As shown in Table 1, there was no statistically significant difference between the VAS scores of the two groups at baseline and 4 h after the surgery. At 8, 12 and 24 h after surgery, the VAS scores of the combination treatment group were significantly lower than those of the control group (8 h: 2.4 vs. 3.0; 12 h: 2.2 vs. 4.2, and 24 h: 2.1 vs. 5.4, respectively, *P* < 0.05 for all comparisons).

**Table 1:** Comparison of VAS score between the two groups

Group	Cases	Preoperative	4 h postoperative	8 h postoperative	12 h postoperative	24 h postoperative
Combination	57	1.7 ± 0.4	1.8 ± 0.9	2.4 ± 0.6^a^	2.2 ± 0.9^a^	2.1 ± 0.4^a^
Control	57	1.8 ± 0.5	1.9 ± 0.7	3.0 ± 1.5	4.2 ± 1.1	5.4 ± 0.8

Note: ^a^t test, *P* < 0.05 compared with the control group

### Ropivacaine in combination with dexmedetomidine exerts significantly longer blocking effects on sensory and motor nerves than ropivacaine alone

The sensory and motor nerve blocking effects in both groups are shown in Table 2. Compared with that of the control group, the time to onset of nerve blockade of both sensory nerves and motor nerves in the combination treatment group was significantly shorter (8.9 min vs. 12.4 min for sensation blockade; 7.5 min vs. 12.8 min for motor blockade, *P* < 0.05 for both comparisons). Furthermore, the blockade maintenance time for both sensory nerve and motor nerve was significantly longer in the combination treatment group than the control group (482.1 min vs. 380.2 min for sensation blockade; 430.1 min vs. 350.1 min for motor blockade, *P* < 0.05 for both comparisons), and the analgesic maintenance time was also significantly longer in the combination treatment group (590.2 min vs.532.1 min, *P* < 0.05).

**Table 2:** Comparison of sensory and motor nerve blocking effects between the two groups

Group	No. of cases	Onset of blockage time (min)		Blockage maintenance time (min)		Analgesia maintenance time (min)
		Motor	Sensation	Motor	Sensation	
Combination	57	7.5 ± 2.3^a^	8.9 ± 2.5^a^	430.1 ± 35.7^a^	482.1 ± 39.4^a^	590.2 ± 40.5^a^
Control	57	12.8 ± 3.7	12.4 ± 2.4	350.1 ± 32.4	380.2 ± 37.2	532.1 ± 36.7

Note: ^a^t test, *P* < 0.05 compared with control group

### Ropivacaine in combination with dexmedetomidine leads to greater improvement in HR, MAP and SPO_2_ than ropivacaine

The vital signs of the study patients at different time points are shown in Table 3. HR, MAP and SPO_2_ exhibited no statistically significant changes after surgery versus baseline in both groups. However, a statistically significant difference was found in HR, MAP and SPO_2_ between the two groups during the surgery: HR and MAP of the combination treatment group were significantly lower than those of the control group (MAP: 78 vs. 84; HR: 72 vs. 79; *P* < 0.05 for both comparisons), and SPO_2_ of the combination treatment group was significant higher than that of the control group (95.1 vs. 88.2; *P* < 0.05), indicating that the combination treatment was more beneficial to the patients.

**Table 3:** Comparison of HR, MAP and SPO2 at different time points between the two groups

Group	Timing	HR	MAP	SPO_2_
Combination	Preoperative	81.0 ± 5.8	84.2 ± 6.5	97.2 ± 3.2
	Intraoperative	72.2 ± 7.4^a^	78.0 ± 5.4^a^	95.1 ± 3.9^a^
	Postoperative	80.2 ± 6.4	84.1 ± 6.2	97.1 ± 3.7
Control	Preoperative	81.5 ± 7.3	84.7 ± 7.1	97.0 ± 4.0
	Intraoperative	79.4 ± 6.8	84.0 ± 5.9	88.2 ± 2.0
	Postoperative	80.0 ± 5.7	84.3 ± 6.8	97.3 ± 4.0

Note: ^a^t test; *P* < 0.05 compared with the control group

### Ropivacaine in combination with dexmedetomidine has significantly fewer adverse reactions than ropivacaine alone

The adverse reactions of the two groups are summarized in Table 4. The major adverse reactions in both groups were lethargy and nausea; however, the incidence rate of adverse reaction in the combination treatment group was significantly lower than that of the control group (3.6 vs. 7.2; *P* < 0.05). However, the cases of the adverse reaction were too few to reach a final conclusion yet, and more studies are needed to verify this.

**Table 4:** Comparison of adverse reactions between the two groups {case (%)}

Group	Cases	Lethargy	Nausea	Incidence rate (%)
Combination	57	1(1.8)	1(1.8)	3.6^a^
Control	57	2(3.6)	2(3.6)	7.2

Note: ^a^χ^2^ test; *P* < 0.05 compared with the control group

## Discussion

This study evaluated the efficacy and safety of ropivacaine in combination with dexmedetomidine versus ropivacaine alone in 114 patients requiring upper limb surgery. The findings showed that ropivacaine in combination with dexmedetomidine was associated with a significantly greater reduction of VAS scores at 8 to 24 h postoperatively compared with ropivacaine alone, suggesting that the combination regimen is more effective than ropivacaine alone in alleviating postoperative pain. We further demonstrated that this improvement in pain reduction was at least partially due to significantly longer blocking effects by the combination therapy on sensory and motor nerves than ropivacaine alone. The study indicates that along with its more benign safety profile, ropivacaine in combination with dexmedetomidine offers a more effective and safer alternative to ropivacaine alone in analgesia for patients undergoing upper limb surgery.

Theoretically, the combined use of two kinds of anesthetics would yield a synergistic effect, leading to a low dosage use of each drug. Several studies have evaluated the dosages and efficacy of the combination of the two anesthetics in different race cohorts [12, 13]. However, few studies on the ropivacaine-dexmedetomidine combination have been reported in Chinese cohorts [14]. In this study, we investigated the analgesic effect of ropivacaine in combination with dexmedetomidine in brachial plexus block and compared its effect with that of ropivacaine alone. The results showed that compared with the control group, nerve blockade onset time in the combination treatment group was significantly shorter, and the blockade maintenance time was markedly longer; the VAS scores of the combination treatment group were significantly lower than that of control group at 8, 12 and 24 h after the operation; furthermore, MAP and HR in combined treatment were significantly lower than those of the control group, and SPO_2_ was significantly higher than that of the control group; the major adverse reactions in both groups were lethargy and nausea, while the incidence rate of adverse reaction in combined treatment group was significantly lower than that of the control group.

Compared with similar studies performed by other investigators [12–14], our results are consistent with their conclusions. Moreover, our study is superior to those already published in several aspects: 1) we had a larger sample size (114 patients in our study compared with 80 patients in the study published). 2) Our study parameters were broader, i.e., VAS score for assessing the effect of analgesia was also included in our study. 3) Detailed study of the adverse reactions was also included in our study.

We found that the major adverse reactions in both groups were lethargy and nausea, and the incidence rate of adverse reaction in the combined treatment group was significantly lower than that of the control group, which is somewhat surprising, as per common sense, one would assume that the combination of two anesthetics would cause more adverse reactions than single anesthetic. For example, Das reported a higher incidence of somnolence in the combination group than the control group [13]. One explanation for the discrepancy is that we used a lower dose of the anesthetics in our study so that they might not cause somnolence in our patient. In the meantime, it is worthy of pointing out that the cases of the adverse reaction in our study were too few (one case vs. two cases) to reach a final conclusion yet, more studies are needed to verify this.

The study also has limitations. The study was undertaken in a tertiary care setting and the findings may not be applicable to primary or secondary care settings. Second, we only evaluated the effect on pain reduction up to 24 h postoperatively. In addition, we only limited the study patients to those undergoing upper limb surgery and the study findings may not be applicable to other types of surgery. Moreover, ultrasonography was not used in the current study as it was not available in our surgical ward.

## Conclusions

To summarize, from this study, it can be concluded that combination use of dexmedetomidine and ropivacaine in the brachial plexus block has a good analgesic effect. Compared with use of ropivacaine alone, the combination regimen has faster nerve blockade onset, longer nerve blocking effect and lower VAS score, it can significantly improve vital signs such as HR, blood pressure and SPO_2_, and can also reduce the incidence of adverse reactions; thus, its application is worth promoting widely.

## Abbreviations

ASA: American Society of Anesthesiologists
HR: heart rate
MAP: mean arterial pressure
SPO_2_: peripheral capillary oxygen saturation
VAS: visual analog scale
